# A combination of Q-TOF LC/MS and LC-MS/MS based metabolomics in pediatric-onset multiple sclerosis demonstrates potential biomarkers for unclassified patients

**DOI:** 10.55730/1300-0144.5436

**Published:** 2022-05-29

**Authors:** İsmail SOLMAZ, Ozan KAPLAN, Mustafa ÇELEBİER, İncilay LAY, Banu ANLAR

**Affiliations:** 1 Department of Pediatric Neurology, Faculty of Medicine, Hacettepe University, Ankara, Turkey; 2 Department of Pediatric Neurology, Faculty of Medicine, University of Health Sciences, Dr. Sami Ulus Maternity and Children’s Health and Diseases Training and Research Hospital, Ankara, Turkey; 3 Department of Analytical Chemistry, Faculty of Pharmacy, Hacettepe University, Ankara, Turkey; 4 Department of Medical Biochemistry, Faculty of Medicine, Hacettepe University, Ankara, Turkey; 5 Clinical Pathology Laboratory, Hacettepe University Hospitals, Ankara, Turkey

**Keywords:** Metabolomics, quadrupole time-of-flight liquid chromatography/mass spectrometry, biomarker, pediatric multiple sclerosis, diagnosis

## Abstract

**Background/aim:**

Metabolomics has the potential to provide putative biomarkers and insights into the pathophysiology and diagnosis of pediatric multiple sclerosis (pMS), which is an inflammatory demyelinating disorder of the central nervous system with a broad spectrum of clinical manifestations. In this study, we aimed to investigate serum metabolomics in pMS to help elucidate the pathophysiology of MS.

**Materials and methods:**

An untargeted approach was applied using the quadrupole time-of-flight liquid chromatography/mass spectrometry (Q-TOF LC/MS) method to study plasma metabolites in patients with pMS (n = 33), patients with unclassified central nervous system demyelinating diseases (n = 6), and age-matched healthy control subjects (n = 40). The patient and control groups were compared for metabolites and the normalized peak areas differed statistically (p < 0.05), showing at least a 1.25-fold change between groups. Bioinformatic tools combined with a clinical perspective were employed for the identification of the putative metabolites. In addition to the untargeted metabolomics approach, targeted LC-MS/MS metabolite analysis was employed to compare the pMS group with the control group.

**Results:**

Significant differences between the patient and control groups were noted for tyramine, 4-hydroxyphenylacetaldehyde, sphingosine/3-dehydrosphinganine, prostaglandins/thromboxane A2, 20-hydroxy-leukotriene E4, 3α,7α,12α-trihydroxy-5β-cholestan-26-al/calcitriol, pantetheine, ketoleucine/3-methyl-2-oxovaleric acid, L-arginine/D-arginine, coproporphyrinogen III, (S)-reticuline, carnosine, cytidine, and phosphoribosyl pyrophosphate. Additional tests for sphingosine 1-phosphate, sphingophosphocholines, ceramides, oxysterols, and calcitriol levels yielded significant metabolomic differences for the pMS group compared to the control group. The metabolomic data of 3/6 patients with unclassified demyelinating disorders matched the pMS group; their follow-up verified the diagnosis of pMS.

**Conclusion:**

In general, plasma metabolites related to sphingolipid metabolism, myelin products, inflammatory pathways, mitochondrial dysfunction, and oxidative stress were found to be altered in cases of pMS. The method applied in this study, combining untargeted analysis with a targeted approach, can be applied to larger series of cases of pMS and other demyelinating disorders for further validation.

## 1. Introduction

Multiple sclerosis (MS) can present with a wide range of neurological symptoms. Its diagnosis is based on clinical evaluation, magnetic resonance imaging (MRI), and cerebrospinal fluid (CSF) analysis. Pediatric multiple sclerosis (pMS) is defined by the first attack occurring before 18 years of age. It constitutes up to 10% of all MS cases and its prevalence ranges between 0.13 and 0.66 per 100,000 children per year [1]. Its differential diagnosis from other inflammatory demyelinating diseases can be difficult and many cases that do not meet the diagnostic criteria of the International Pediatric Multiple Sclerosis Group (IPMSSG) [2] remain unclear. Disease-related biomarkers might assist in such cases and may also provide information on the pathogenesis, treatment, and prognosis of the disease.

Metabolomics is a powerful tool in the investigation of putative biomarkers. It entails the dynamic, comprehensive, large-scale analysis of small molecules downstream of the proteome and, therefore, closer to the phenotype of an organism in comparison to genomics, transcriptomics, or proteomics. The metabolome comprises more than 15,000 endogenous metabolites affected by diseases. Although no technique is capable of analyzing the whole metabolome with a single injection, metabolomic research techniques for sample preparation, analysis, computerized interpretation, and data banks are constantly being improved [3].

The Human Metabolome Database (HMDB, www.hmdb.ca) is a web-enabled database containing comprehensive information about human metabolites and their biological roles, physiological concentrations, disease associations, chemical reactions, metabolic pathways, reference spectra, interactions with single-nucleotide polymorphisms, and the effects of drugs on these metabolites. In the last decade, the HMDB was expanded from 6408 to 114,100 metabolites (http://www.hmdb.ca/statistics) and it is now considered the standard resource for human metabolomic studies [3]. Databases allow the interpretation of untargeted metabolomic studies using advanced analytical techniques such as quadrupole time-of-flight liquid chromatography/mass spectrometry (Q-TOF LC/MS). However, a limitation of untargeted studies is the possibility of one mass/charge (m/z) ratio matching with more than one metabolite. Such situations can be overcome with recent data-mining strategies using platforms like MetaboAnalyst, which allow users to match the final m/z list of the peaks with metabolites logically using pathway analysis [4]. Furthermore, examining the final data from a clinical perspective can be useful for disregarding irrelevant results.

In this study, plasma samples of patients with pMS diagnosed according to clinical and radiological criteria, patients with unclassified central nervous system (CNS) demyelinating diseases, and healthy control subjects were studied. An untargeted Q-TOF LC/MS metabolomics approach and, where applicable, a targeted LC-MS/MS approach were applied to elucidate metabolites specific to pMS. The raw data of the untargeted study [5] were processed with MetaboAnalyst. The results were examined for metabolites likely to be involved in the pathogenesis of the disease. This is one of the first metabolomic studies of pMS using this approach.

## 2. Materials and methods

### 2.1. Study population and sample collection

This research was undertaken with a single-center, prospective design. The diagnosis of pMS was made according to IPMSSG criteria [2]. Patients with pMS (Group pMS, n = 33), patients with an unclassified CNS demyelinating disorder not matching the diagnostic criteria of pMS [Group unknown (Group U), n = 6], and a control group (Group C, n = 40) in which inflammatory demyelinating disorders ruled out were included in the study. Sampling was done when patients came to routine control visits or were hospitalized during an attack. Those in Group pMS who received beta interferon (β-IFN) treatment were divided into two subgroups to evaluate the metabolomic differentiation between relapse and remission periods as Group pMS-T1 and Group pMS-T2. Sampling for Group pMS-T1 (n = 10) was done in the remission period, while for Group pMS-T2 (n = 5), sampling was done in the relapse period [not all patients with pMS who received β-IFN (n = 20) could be included in Group pMS-T_1_ or Group pMS-T_2_]. Since pooled samples were used to compare the pMS and control groups, individual plasma samples of pMS patients were selected randomly from among the pooled pMS samples to form Group Random (Group R, n = 6) to verify whether the pooled pMS samples represented the pMS metabolome. This internal control process was verified using principal component analysis (PCA) graphs. Demographic and clinical data are summarized in Table 1. All patients and control subjects were examined in the Hacettepe University, Faculty of Medicine’s Department of Pediatric Neurology. Informed consent from all patients and parents and ethical approval from the institution (2019/21-06) were obtained.

### 2.2. Preparation of the samples

**Figure 1:**
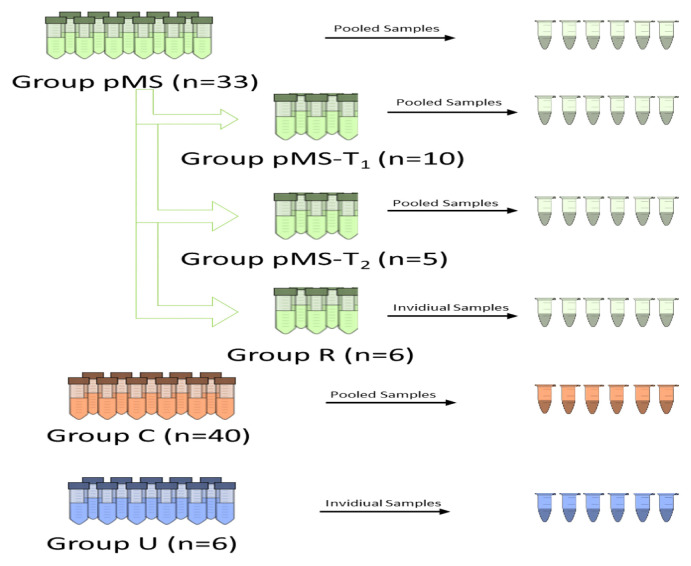
An illustration of sample collection and preparation.

Plasma was separated from 3 mL of peripheral venous blood from each participant and withdrawn into tubes containing ethylenediaminetetraacetic acid (3000 rpm, 10 min). The plasma samples were held at –80 °C until analysis. Stored samples were thawed on the day of the experiment and 200 μL of plasma from each patient from Group pMS including both pMS-T_1_ and pMS-T_2_ were pooled in conical Falcon tubes of 15 mL (Fisher Scientific, Portsmouth, NH, USA) and vortexed. Samples from Group C were pooled in the same way. The samples from Group R and Group U were prepared as individual samples. All pooled and individual samples were subjected to metabolite extraction by nanofiltration using ultrafiltration cartridges (3 kDa pores, Amicon Ultra 0.5 mL Centrifugal Filters, Merck, Darmstadt, Germany), and 300 μL of sample and 150 μL of acetonitrile were placed into a cartridge and centrifuged (15,000 rpm, 45 min). The filtered liquid (metabolite phase) was collected and dried with a vacuum centrifuge (9 °C, Labconco CentriVap Benchtop Vacuum Concentrator, Kansas City, MO, USA), then dissolved with acetonitrile and water (300 μL, 1:1 v/v). All solvents were of LC-MS grade (Sigma-Aldrich, St. Louis, MO, USA). Pooled samples of Group pMS and Group C were subjected to consecutive dilutions (dilution factors: 4:4, 4:4, 4:4, 3:4, 2:4, 1:4 v/v) as described in the literature to process the data statistically and prevent false-positive peaks [6]. Figure 1 illustrates the steps of sample collection and preparation.

### 2.3. Untargeted and targeted metabolomics

#### 2.3.1. Q-TOF LC/MS analyses

The experiments were performed on an Agilent 6530 LC/MS Q-TOF instrument (Agilent Technologies, Santa Clara, CA, USA) using a C18 column (Agilent Zorbax 1.8 μM, 50.0×2.1 mm). Mobile phases (acetonitrile:water, 0.1% formic acid) were subjected to a flow of 0.20 mL min^–1^ that began with 90% H_2_O until the end of the 1^st^ min and then the acetonitrile ratio was increased linearly to 90% by the 15^th^ min. The chromatographic conditions were then linearly returned to the starting conditions by the 20^th^ min and a 5–min post-run was applied for further injections. The aim of using this gradient elution program was to separate both polar and nonpolar metabolites properly under the selected chromatographic conditions. The scan range for the MS device was 100–1700 m/z. All samples were injected as two replicates in random order. The column temperature was 30 °C and capillary voltage was 4000 V. The mass spectrometer, equipped with an ESI probe, was operated in positive (ESI+) ion mode using the standards recommended by the manufacturer for the device. System suitability for LC separation and MS detection was measured using quality control (QC) samples (Waters Co., LC/MS QC Reference Standard [186006963]), which were injected before the analysis. Injection repeatability was checked with retention time and peak intensity shifts. The reproducible chromatographic conditions allowed the performance of all experiments under identical conditions.

#### 2.3.2. Data processing

Raw chromatograms for all groups were processed with XCMS using parameters optimized with the Isotopologue Parameter Optimization (IPO) tool [7]. A normalization procedure was performed before the statistical comparison of groups. False-positive peaks were eliminated from pooled samples (Group pMS and Group C) with the application of recently introduced data processing strategies including a consecutive dilution technique to eliminate false-positive peaks and peak normalization to discard random errors from peak areas [6]. Peaks normalized using the total area of all peaks were compared among themselves and the peaks with significant differences (p < 0.05) and fold change values of >1.25 between Groups pMS and C were statistically compared with the individual samples (Groups U and R). Normalizations and comparisons of peak areas were performed with MS Excel and t-tests were used for statistical comparison. The MS Peaks to Pathways utility of MetaboAnalyst 4.0 was used to match the final list of peaks with Kyoto Encyclopedia of Genes and Genomes (KEGG) codes to identify potential biomarkers, which were then reviewed with a clinical evaluation using a detailed literature search to associate the results with the pathology of the disease. In addition, the results were visualized using two-dimensional PCA score plots, as is commonly done for large volumes of data from high-throughput metabolomics experiments.

#### 2.3.3. LC-MS/MS analyses

In addition to the untargeted metabolomics approach, some potential biomarkers related to putative metabolites found in untargeted analysis were also measured with a targeted approach. Sphingosine 1-phosphate, long-chain (16–20) and very long-chain (22–24) ceramide, and ceramide phosphocholine species were quantified using internal standards by LC-MS/MS according to a previously applied method [8]. Optimized multiple reaction monitoring with positive electrospray ionization (ESI) was used in ultrafast liquid chromatography (LC-20 AD UFLC XR, Shimadzu Corporation, Kyoto, Japan) coupled with MS-8040 triple quadrupole mass spectrometry (Shimadzu Corporation). Chromatographic separations were carried out using an HPLC column (XTerra 18, 2.1 × 50 mm, Waters, Milford, MA, USA). 7-Ketocholesterol and cholestane-3β,5α,6β-triol were quantified with N,N-dimethylglycine derivatization in a modified application of the LC-MS/MS method (MS-8040, Shimadzu Corporation) [9]. Chromatographic separations were done using a Symmetry C18 column (100 × 2.1 mm, 5 μm) (Thermo Fisher Scientific, Waltham, MA, USA). Calcitriol was also quantified by LC-MS/MS (MS-8040, Shimadzu Corporation).

## 3. Results

### 3.1. List of metabolites changed in pMS

**Figure 2:**
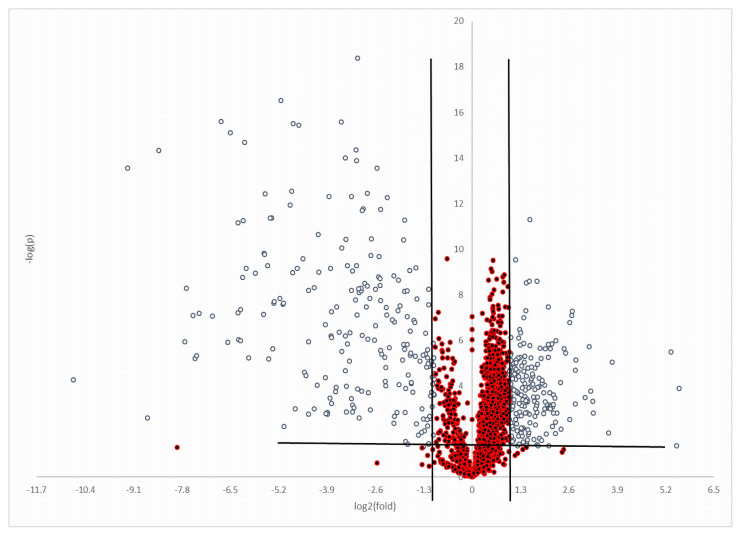
Volcano plot showing the distribution of the detected metabolite peaks for groups pMS and Group C. Gray dots: The metabolites examined in this study (fold change of >1.5, p < 0.05).

Metabolites differing between pMS and healthy control (Group C) samples were identified; most peaks were eluted after 14 to 26 min. No marked difference was observed between the chromatograms of Groups pMS and C. The number of peaks detected with the optimum IPO parameters was 2371. The remaining 907 peaks after data processing [6] were examined for statistical differences between the groups and the peaks showing a fold change of >1.25 and p < 0.05 between Groups pMS and C (n = 349) are presented in a volcano plot in Figure 2. The remaining list was processed via MetaboAnalyst 4.0 for putative identification and the results were evaluated based on a literature search to associate the metabolites with the disease. Finally, 23 metabolites corresponding to 14 peaks were found to be important (Table 2). Measurements of sphingosine 1-phosphate, sphingophosphocholines, ceramides, 7-ketocholesterol, cholestane-3β,5α,6β-triol, and calcitriol levels showed changes in the same direction with untargeted data, although not differing significantly between groups (Table 3).

The combination of the untargeted results with the targeted results indicated increased levels of sphingosine-1-phosphate, tyramine, 4-hydroxyphenylacetaldehyde, pantetheine, 7-ketocholesterol, and cholestane-3β,5α,6β-triol and decreased levels of eicosanoids, sphingosine/3-dehydrosphinganine, coproporphyrinogen III, (S)-reticuline, ketoleucine/3-methyl-2-oxovaleric acid, L-arginine/D-arginine, carnosine, 3α,7α,12α-trihydroxy-5β-cholestan-26-al/calcitriol/sarsasapogenin, 20-hydroxy-leukotriene E4, phosphoribosyl pyrophosphate, long-chain (18–20) and very long-chain (22–24) ceramides, and ceramide phosphocholines in Group pMS compared to control samples (Group C).

### 3.2. PCA results

**Figure 3:**
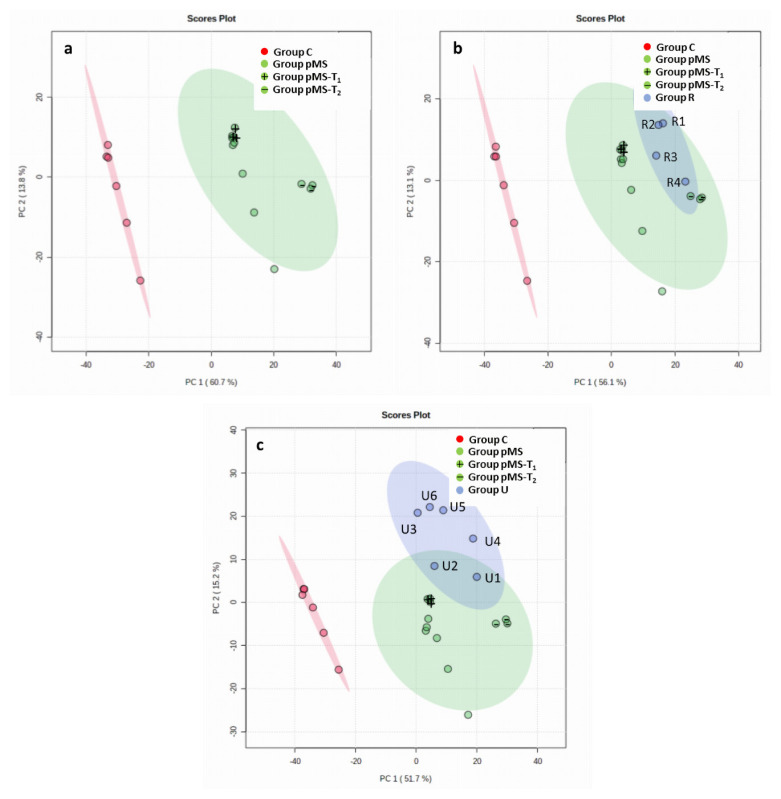
Principal component analysis graphs. **Distribution of the metabolite profiles of the groups**. a) Groups pMS, pMS-T_1_, and pMS-T_2_ vs. Group C; b) Groups pMS, pMS-T_1_, pMS-T_2_, and R vs. Group C; c) Groups pMS, pMS-T_1_, pMS-T_2_, and U vs. Group C.

The PCA results showed that Group pMS and its subgroups (i.e. Group pMS-T_1_ and Group pMS-T_2_) were distinguishable from Group C (Figure 3a). The metabolite profiles of Group pMS-T1 and Group pMS-T2 did not differ statistically from Group pMS or from each other. The individual samples of Group R, used to validate the results, were found to constitute a subset of the cluster formed by Group pMS, Group pMS-T_1_, and Group pMS-T_2_ (Figure 3b). This shows that the metabolite profiles of the individual samples of Group R are represented by the pooled samples of Group pMS. Group U samples were statistically different from Group C; individual samples U3, U4, U5, and U6 were outside of the cluster formed by Groups pMS, pMS-T_1_, and pMS-T_2_ (Figure 3c).

## 4. Discussion

Demyelinating diseases constitute a large group including MS, neuromyelitis optica, acute disseminated encephalomyelitis, and neuromyelitis optica spectrum disorder (NMOSD), and their treatment approaches differ considerably. However, distinctions within this spectrum can be difficult and certain cases do not receive a definite diagnosis by clinical and imaging findings at the time of the initial episode. Therefore, biomarkers reflecting metabolic status are being investigated to assist in clinical decisions; they can also clarify pathophysiology.

### 4.1. Proposed biomarker set based on results

Previous plasma metabolic profiling by nuclear magnetic resonance spectroscopy and PCA graph analysis for 108 individuals with demyelinating disorders (34 with relapsing-remitting MS, 54 with AQP4-Ab NMOSD, and 20 with MOG-Ab disease) suggested that relapsing-remitting MS could be differentiated from aquaporin-4 IgG-positive NMOSD through various combinations of scyllo-inositol, myo-inositol, lipoprotein particles, histidine, glucose, lactate, alanine, formate, and leucine that differed between the groups [10]. Another study showed that higher levels of myoinositol and formate in relapsing-remitting MS allowed differentiation from antibody-negative NMOSD [11]. Scyllo-inositol and myo-inositol are metabolites of sphingosine metabolism. In our study, plasma sphingosine was decreased and sphingosine-1-phosphate (S1P) was elevated in Group pMS compared to Group C. In MS, sphingolipids released from damaged myelin can be broken down to sphingosine, which then turns into S1P in states of intense inflammation or immune dysregulation [12]. MS-associated proinflammatory cytokines can activate the sphingomyelin cycle in oligodendrocytes. Studies found higher S1P levels in the CSF and lower sphingosine and dihydrosphingosine levels in the blood of MS patients compared to a control group [13].

Ceramides are mainly generated by the hydrolysis of sphingomyelin, but they are also synthesized de novo and converted to complex sphingolipids. They take part in the myelin structure and signal transduction, and they have bioactive roles in inflammation and neurodegeneration. Group pMS had reduced long-chain (18–20) and very long-chain (22–24) ceramides, but not C16 ceramide, and reduced 16–24 sphingomyelins (ceramide phosphocholine) compared to Group C. Kurz et al., in a cohort study that included 72 MS patients and 25 healthy controls, demonstrated downregulated C24 ceramide in the leukocytes of MS patients and upregulated C16 and C24:1 ceramides in control plasma [14]. Interestingly, they found that C16-LacCer was downregulated in MS patients compared to healthy controls; likewise, only C16 ceramide was found to be higher in pMS patients in our study. Therefore, low levels of long-chain (18–20) and very long-chain (22–24) ceramides, 16–24 sphingomyelins, and sphingosine and high levels of S1P suggest both myelin alteration or degradation and the role of sphingosine and ceramide metabolism in the pathophysiology of MS.

Tyramine is a naturally occurring trace amine derived from tyrosine. It was significantly increased in Group pMS (Group pMS/Group C ratio: 14.38) in the present study, and 4-hydroxyphenylacetaldehyde, produced from tyramine by monoamine oxidase and from L-tyrosine by activated phagocytes, was also elevated by 1.69-fold. Tyramine promotes the release and actions of adrenergic transmitters, which act on immune and neural cells, as reported in cases of MS [15].

Pantetheine, a cysteamine amide analog of pantothenic acid (vitamin B5), was increased by 2.38-fold in Group pMS. Pantetheine is an intermediate in the biosynthesis of coenzyme A, which has an important role in energy metabolism, especially in mitochondrial reactions; its deficiency can potentiate oxidative stress, inflammation, and demyelination [16]. In MS patients, the disturbance of pyruvate metabolism and mitochondrial reactions can increase extramitochondrial glucose and anaerobic metabolites [17].

Prostaglandins or thromboxane A2 are involved in many inflammatory disorders. The main enzyme in their synthesis from arachidonic acid is 5-lipoxygenase, which is upregulated in peripheral blood cells of MS patients during relapse. The action of 5-lipoxygenase varies according to its intracellular localization; 5-lipoxygenase on the nuclear membrane of neutrophils, mast cells, and macrophages promote the synthesis of proinflammatory leukotriene B4 while cytoplasmic 5-lipoxygenase favors the synthesis of the regulating mediator lipoxin A4 in macrophages [18]. Likewise, some prostaglandins are pro- and others are antiinflammatory. Prostaglandin-E1 and D2 have been implicated in the treatment of MS, both within the action of certain drugs and as separate therapeutic options [19]. Thromboxane production has been studied in progressive but not relapsing-remitting MS. Its detection in plasma indicates the activation of endothelia or monocytes in an inflammatory state [20]. The lower levels observed in Group pMS compared to Group C in the present study may imply the role of eicosanoids in pMS, but this needs verification by targeted measurements of individual eicosanoids.

(S)-Reticuline, an endogenous precursor of morphine, was lower in Group pMS. Morphine in human tissue is considered to be of exogenous origin, but recent studies have shown that human cells can produce morphine in nanomolar ranges and (S)-reticuline is an important precursor [21]. Thus, the decreased (S)-reticuline in Group pMS may imply decreased synthesis of morphine at the metabolite level. Morphine has immunomodulatory effects in humans through brain-like-opioid receptors expressed on lymphocytes [22]. The effect of endogenous morphine in the immunopathogenesis of MS should be explored further.

Coproporphyrinogen III, a porphyrin metabolite arising from heme synthesis, was lower in Group pMS compared to control samples, which may reflect diminished synthesis. Heme is found in a number of hemoproteins such as hemoglobin, myoglobin, cytochromes, catalases, heme peroxidase, and endothelial nitric oxide synthase (NOS). Free hemoglobin can contribute to MS pathogenesis by damaging the blood-brain barrier and myelin. However, hemoglobin in neural cells is considered to be neuroprotective [23]. An important heme-containing complex is the ubiquinone-cytochrome c reductase in the mitochondria; reduced function of this complex may result in mitochondrial dysfunction [24]. Therefore, reduced heme synthesis may contribute to cellular damage.

Ketoleucine and 3-methyl-2-oxovaleric acid are abnormal metabolites arising from the incomplete breakdown of branched-chain amino acids. Lower levels were detected in Group pMS compared to controls. Noga et al., in a rat model of MS, observed that arginine, alanine, and branched amino acids were low at the time of onset and increased at the peak of the disease [25]. As branched-chain amino acids are a source of pyruvate for energy metabolism or de novo synthesis of macromolecules, their reduction may suggest utilization within neural and immune cells in early-stage pMS [26].

The peaks of D- and L-arginine were significantly smaller in Group pMS (Group pMS/Group C ratio: 0.59). Arginine is converted by NOS to NO, which can be neuroprotective or neurotoxic depending on the degree of injury, cellular redox status, location, and concentration of synthesis. NO is associated with oligodendrocyte dysfunction in demyelinating diseases [27]. NO synthesis is stimulated by activated microglia and Th17 cells and it may be increased in the tissues, CSF, and plasma of MS patients. Therefore, reduction of arginine may be expected [28]. A study comparing the plasma metabolomics of 28 patients with relapsing-remitting MS and 18 unaffected individuals found lower concentrations of arginine in the MS group, which also correlated with certain MRI findings [29].

Carnosine is a naturally distributed dipeptide composed of histidine and β-alanine whose modulation of free radical production results in antioxidant, antiinflammatory, antiapoptotic, and particularly neuroprotective properties [30]. In Group pMS in the present study, the low level of carnosine (Group pMS/Group C ratio: 0.57) might indicate the contribution of similar mechanisms.

Diminished levels of 3α,7α,12α-trihydroxy-5β-cholestan-26-al, an intermediate in bile acid biosynthesis, and elevated levels of 7-ketocholesterol and cholestane-3β,5α,6β-triol were found in Group pMS. Bhargava et al. reported that MS and pMS patients had lower levels of bile acid metabolites [31]. Bile acids may be neuroprotective and antiinflammatory in neurodegenerative disorders as enzymes of the alternative bile acid synthesis pathway are expressed in the CNS. Circulating bile acids can act on receptors expressed on astrocytes and macrophage/microglia, and supplementation was found to be of benefit in experimental autoimmune encephalomyelitis [31, 32]. Therefore, oxysterols might be involved in pMS within inflammatory pathways.

Calcitriol, the physiologically active form of vitamin D, is known to play a preventive role in MS [33]. Numerous studies on sunlight exposure, latitude, and diet support a correlation between serum concentrations of vitamin D and disease severity [34]. As expected, our pMS patients had lower calcitriol levels as confirmed by targeted analysis (Group pMS/Group C calcitriol ratio: 0.86).

Decreased phosphoribosyl pyrophosphate in Group pMS implies inadequate transfer of phosphate groups in the biosynthesis of histidine, tryptophan, and purine-pyrimidine nucleotides. Tryptophan and its metabolites affect immune response in MS; their levels were low in adult MS patients compared to healthy subjects. In addition, higher serum levels of tryptophan were associated with a lower risk of pMS [35]. Histidine, another amino acid reported to be neuroprotective, was also low in serum samples from MS patients [36]. Purinergic signals play key roles in myelination, neuroprotection, and neurodegeneration, and they have been implicated in neurodegenerative and neuroinflammatory diseases [37]. Reduced levels of tryptophan, histidine, purine, and pyrimidine may take part in neuroprotection and inflammatory regulation in pMS.

Cytidine is a pyrimidine incorporated into nucleic acids and it can serve as a substrate for the salvage pathway of pyrimidine nucleotide synthesis. Cytidine triphosphate is involved in the biosynthesis of phosphatidylcholine and phosphatidylethanolamine, the most abundant phosphatides in the brain. Together with studies on phospholipids in MS, low cytidine levels in Group pMS in the present study support a pathogenetic role of phosphatidylcholine [38].

### 4.2. PCA results for monitoring pMS

The PCA results showed that Group pMS and its subgroups (pMS-T_1_ and pMS-T_2_) were distinguishable from Group C. Individual random samples (Group R) used to validate the results fell within the cluster formed by Groups pMS, pMS-T_1_, and pMS-T_2_. As expected, Group U samples differed from those of Group C. Upon follow-up, U3, U5, and U6 were diagnosed with pMS; U1 was diagnosed with antimyelin oligodendrocyte glycoprotein (MOG) disease; U2 retained the diagnosis of clinically isolated syndrome (CIS); and U4 was diagnosed with parainfectious myelitis, indicating classification errors in the cases of U3, U4, and U6. The PCA graphs (Figure 3) illustrate this approach, as individual random samples (Group R) could easily be distinguished from control samples, unclassified samples that turned out to be non-MS (U1, U2) could be categorized correctly, and U3, U4, U5, and U6 were erroneously categorized outside of the pMS group. Although the random samples (Group R) corresponded with the cluster of Group pMS, U3, U4, U5, and U6 did not meet the diagnostic criteria of pMS while the analysis was being performed. This suggests that even if metabolomic analysis reflects the actual conditions clearly, it has some limitations in suggesting further predictions for the diagnosis of pMS. Another important point illustrated by the PCA graphs was the similar distribution of pMS-T_1_ and pMS-T_2_, suggesting that clinical attacks and β-IFN treatment make no detectable differences at this metabolomic level.

The pathogenesis of MS involves numerous inflammatory reactions that have been studied with a variety of methods. To date, however, there is no candidate biomarker that can point to the disease mechanism in MS.

This first untargeted metabolomic study of pMS has indicated pathways involving sphingolipid, branched-chain amino acid, and bile acid metabolism along with mitochondrial dysfunction and oxidative stress.

The use of pooled samples rather than individual samples minimizes individual metabolomic changes and the metabolites found to differ between pooled pMS and control groups are those directly related to pMS. This type of analysis is complemented by comparison of individual samples from subjects with and without pMS, allowing the evaluation of metabolites that differ in individual random samples.

The limitations of this study include the sizes of Group pMS and its subgroups. Although these were comparable to group sizes of the previous studies cited above and acceptable considering the prevalence of pMS, further studies with larger series reflecting various stages of the disease are needed. The sizes of the other groups were also limited. In particular, expanding the category of “unclassified demyelinating diseases” is important for clinical applications of this method. The “unclassified” group was named as such at the time of plasma sampling; some of those individuals received diagnoses of CIS, isolated myelitis, and MOG Ab-related disease upon follow-up. The search for related biomarkers specifically aims to avoid diagnostic delays such as these. The duration of disease was short for most of our patients, as can be expected in cases of pMS. Although a limitation in general, this can also be an advantage, as the chronicity and severity of a clinical condition can affect metabolomics and our findings may reflect the early stages, where inflammation is prominent and neurodegeneration is mild. The examination of changes that develop over the course of the disease through longitudinal studies and correlations of metabolomic findings with MRI and Expanded Disability Status Scale (EDSS) results would be of scientific and clinical interest. Likewise, larger numbers of patients with other CNS demyelinating disorders and pMS patients receiving various disease-modifying treatments need to be compared. As our study was primarily designed for large-scale metabolomic screening, targeted analyses were very limited; in the future, such studies should include all metabolites of interest. On the other hand, important strengths of this study include the demonstration of metabolomic changes allowing clear separation between the definite pMS and control groups, and the findings for the test group of “unclassified” demyelinating diseases being similar to those of Group pMS. These initial results obtained from untargeted metabolomic analysis are encouraging for further research investigating the application of these methods in the diagnosis, differential diagnosis, and prediction of pMS.

## Figures and Tables

**Table 1:** Demographic data and distribution of groups.

	Group pMS (n = 33)	Group U (n = 6)	Group C (n = 40)	Group T (n = 15)	Group pMS-T_1_ (n = 10)	Group pMS-T_2_ (n = 5)
**Gender**	26F, 7M	3F, 3M	31F, 9M	12F, 3M	8F, 2M	4F, 1M
**Age at blood sampling (years)**	16.4 ± 2.7 (range: 11–22, median: 17)	12.1 ± 4.9 (range: 3–16, median: 14.5)	16.2 ± 2.7 (range: 9–22, median: 16.5)	16.5±2 (range: 13–19)	17.4 ± 1.6 (range: 14–19)	14.8 ± 1.6 (range: 13–17)
**Age at diagnosis**	13 ± 2.7 (range: 4–17, median: 14)	12.8 ± 4.7 (range: 4–16, median: 15)		13.2 ± 3 (range: 4–17, median: 14)	13.7 ± 1.8 (range: 10–17, median: 13.5)	12.2 ± 4.9 (range: 4–17, median: 14)
**Time from diagnosis toblood sampling (years)**	3.5 ± 2.6 (range: 0.5–10, median: 4)	1.1 ± 0.2 (range: 1–1.5, median: 1)		3.5 ± 2.6 (range: 0.5–10, median: 4)	3.7 ± 1.6 (range: 1–6, median: 4)	3.1 ± 4.1 (range: 0.5–10, median: 0.5)
**Oligoclonal band positivity**	26/29 (89.6%)	4/6 (33%)				
**Attack status at the time of sampling**	8/33 patients (24%) within a week of relapse (3/8 under treatment); others in remission under treatment	4/6 within a week of the episode	-			All within a week of the episode
Treatment	β-IFN:20, GA:1, Fg:4, DMF:4, NTZ:1, TRF:1, NT:2	None	-	β-IFN	β-IFN	β-IFN

**Abbreviations: Group pMS:** Group with pediatric multiple sclerosis, **Group U:** Group with unknown diagnosis, **Group C:** Control group, **Group T:** 15 of 20 patients receiving β-IFN (Group T: Group pMS-T_1_ plus Group pMS-T_2_), **Group pMS-T_1_:** 10 patients in remission from Group pMS-T who received β-IFN, **Group pMS-T_2_:** 5 patients from group pMS-T who received β-IFN and were in relapse, **β-IFN:** Beta-interferon, **M:** Male, **F:** Female **GA:** Glatiramer acetate **Fg:** Fingolimod **DMF:** Dimethyl fumarate **NTZ:** Natalizumab **TRF:** Teriflunomide **NT:** No treatment

**Table 2:** Putative identification of 14 peaks found to be statistically different and having fold changes of >1.25 between Group pMS and Group C.

Peak #	KEGG Code	HMDB Code	Identity	pMS/C
**1**	C00233	HMDB0000695	Ketoleucine	0.60
	C03465	HMDB0000491	3-Methyl-2-oxovaleric acid	
**2**	C03765	HMDB0003767	4-Hydroxyphenylacetaldehyde	1.69
**3**	C00483	HMDB0000306	Tyramine	14.38
**4**	C00062	HMDB0000517	L-Arginine	0.59
	C00792	HMDB0003416	D-Arginine	
**5**	C00386	HMDB0000033	Carnosine	0.57
**6**	C00475	HMDB0000089	Cytidine	0.29
**7**	C00831	HMDB0003426	Pantetheine	2.38
**8**	C00319	HMDB0000252	Sphingosine	0.62
	C02934	HMDB0001480	3-Dehydrosphinganine	
**9**	C03263	HMDB0001261	Coproporphyrinogen III	0.61
**10**	C00427	HMDB0001381	Prostaglandin H2	0.80
	C00584	HMDB0001220	Prostaglandin E2	
	C00696	HMDB0001403	Prostaglandin D2	
	C01312	HMDB0001335	Prostaglandin I2	
	C02198	HMDB0001452	Thromboxane A2	
**11**	C02105	HMDB0003601	(S)-Reticuline	0.61
**12**	C00119	HMDB0000280	Phosphoribosyl pyrophosphate	0.30
**13**	C03577	HMDB0012639	20-Hydroxy-leukotriene E4	0.53
**14**	C01301	HMDB0003533	3α,7α,12α-Trihydroxy-5β-cholestan-26-al	0.57
	C01673	HMDB0001903	Calcitriol	
	C03963	HMDB0030024	Sarsasapogenin	

**Table 3:** Results of targeted analyses.

Group/metabolite	Metabolite	pMS/C
**Sphingolipids (μg/mL)**	16:0 SM (d18:1/16:0)	0.86
	18:0 SM (d18:1/18:0)	0.56
	24:0 SM (d18:1/24:0)	0.68
	S1P	1.35
**Ceramides (ng/mL)**	C16 CER (d18:1/16:0)	1.14
	C18 CER (d18:1/18:0)	0.73
	C20 CER (d18:1/20:0)	0.69
	C22 CER (d18:1/22:0)	0.75
	C24 CER (d18:1/24:0)	0.94
**Calcitriol (pg/mL)**		0.86
**7-ketocholesterol (7-KC) (ng/mL)**		1.02
**Cholestane-3β,5α,6β-triol (chol-triol) (ng/mL)**		**1.47**

**Abbreviations:** 16:0 SM (d18:1/16:0), N-palmitoyl-D-erythro-sphingosylphosphorylcholine.

18:0 SM (d18:1/18:0), N-stearoyl-D-erythro-sphingosylphosphorylcholine.

24:0 SM (d18:1/24:0), N-lignoceroyl-D-erythro-sphingosylphosphorylcholine.

S1P, Sphingosine-1-phosphate.

C16 CER (d18:1/16:0), N-palmitoyl-D-erythro-sphingosine.

C18 CER (d18:1/18:0), N-stearoyl-D-erythro-sphingosine.

C20 CER (d18:1/20:0), N-arachidoyl-D-erythro-sphingosine.

C22 CER (d18:1/22:0), N-behenoyl-D-erythro-sphingosine.

C24 CER (d18:1/24:0), N-lignoceroyl-D-erythro-sphingosine.
